# The Fatal Defects in Cast Al-Si Alloys Due to Sn Addition

**DOI:** 10.3390/ma16176020

**Published:** 2023-09-01

**Authors:** Yao Xiao, Jicheng Wang, Qianyu Deng, Li Feng, Dianming Peng, Hui Feng, Kai Li, Yong Du

**Affiliations:** 1 State Key Laboratory of Powder Metallurgy, Central South University, Changsha 410083, China; xiaoyao0566@csu.edu.cn (Y.X.); yong-du@csu.edu.cn (Y.D.); 2 Runxingtai Electric Appliance Co., Ltd., Zhuhai 519000, China; jichengwang@yc-dc.com (J.W.); dqydengqy@163.com (Q.D.); fengli@frd.cn (L.F.); pengdianming@frd.cn (D.P.); 3 Research Institute for Aerospace Manufacturing Technology, Nanjing Chenguang Group Co., Ltd., Nanjing 210006, China; fenghuihui22@126.com; 4 Hunan Center for Electron Microscopy, Central South University, Changsha 410083, China

**Keywords:** Al-Si alloys, cast defect, Sn, Sn oxides, FIB, HADDF-STEM

## Abstract

Cast defects are common in cast alloys and they are difficult to eliminate without deformation. They strongly degrade the mechanical properties of cast alloys. The addition of some elements can affect the number of cast defects. In this work, the deleterious effect of Sn addition on the mechanical properties of Al-Si alloys has been investigated via 3D-computed tomography, SEM and TEM. Amorphous Sn oxides were found near the alumina film or formed enclosures with alumina film. The melt containing high Sn content was trapped by enclosures, causing more shrinkage pores during solidification. Cracks likely initiated and expanded along these pores and brittle amorphous Sn oxides, deteriorating the mechanical properties. This work suggests not adding Sn to various Al alloys when used in a cast state.

## 1. Introduction

The unibody casting technology of car manufacturing initiated a major revolution in the die-cast aluminum alloy industry [1]. To minimize cost, heat treatments using this technology are cancelled. Among all aluminum alloys, Al-Si alloys with outstanding castability are the most suitable for use with this technology. Casting defects like shrinkages, pores and oxides, are common in Al-Si alloys and strongly degrade their mechanical properties [2]. However, unlike wrought Al alloys such as the 2000, 6000 and 7000 series [3,4,5,6], casting defects of Al-Si alloys cannot be reduced through deformation processes. Therefore, it is very important to strictly control these casting defects.

Besides the parameters of casting [7,8,9], compositions also strongly affect casting defects in Al-Si alloys. For example, the plate-like β-AlFeSi is always generated in Al-Si alloys with Fe addition, which increases porosity during solidification by blocking liquid feeding channels [10,11,12]. The addition of Mn can transform β-AlFeSi phases into Chinese script such as α-AlFeMnSi phases [11,13,14,15], contributing to the reduction in porosity. Additionally, even though Sr can effectively modify Si phases in Al-Si alloys [16], more hydrogen pores are likely to be introduced into melts due to their high tendency to absorb hydrogen [17]. 

In recent years, the strengthening effects of Sn have been found in various wrought and cast Al alloys [18,19,20,21,22,23,24,25]. The mechanism has been revealed by the work of Elsayed et al. [25] on a model Al-0.025 at.% Sn cast alloy. They found that Sn solutes effectively trap and release vacancies during quenching and heat treatments, respectively. Therefore, when Sn was added to age-hardenable alloys such as Al-Cu, the θ′ precipitates were likely to finely distribute in a high-number density in subsequent heat treatments. Similarly, in the cast Al-7 wt.% Si-7 wt.% Cu alloy studied by Akopyan [24] et al., the addition of 0.2 wt.% Sn was found to promote the precipitation of θ′ and θ″ during heat treatments and stabilize the precipitates. The mechanism is also active in wrought Al-Mg-Si-Sn alloys [18]. The research on the effect of Sn addition to cast Al-Si alloys can also provide a basis for designing composites of these alloys [26]. 

However, a strengthening effect of Sn addition onAl-Si cast alloys was rarely observed. Kozana et al. [27] inferred that additions of 0.2–1.7 wt.% Sn to an Al-10Si alloy caused a decrease in the solidification points of α-Al according to their thermodynamic calculations, leading to a drop in melt undercooling and microstructure refinement. As a result, the alloy’s ultimate tensile strength was decreased. Qiu [28] et al. also found that Sn addition led to a decrease in the hardness of T6-treated A356 alloys by nearly 25%. The impairing effect of Sn was attributed by them to the generation of soft β-Sn phases, and the fact that the strengthening phase Mg_2_Si was replaced by Mg_2_Sn. Zhou [29] et al. found some Sn-segregated particles surrounding black pores. However, they did not analyze the relationship deeply between defects and Sn addition. In this paper, the deteriorating effect of Sn on the mechanical properties of Al-Si cast alloys, which related to cast defects, was found at multi-scales via 3D-computed tomography, SEM and TEM.

## 2. Materials and Methods

The Al-9Si and Al-9Si-0.1Sn alloys were prepared, and the chemical compositions of these alloys are shown in Table 1. The alloys were designed as the casting Al alloys for automobiles, which a usually used in the body or chassis of the cars. The effect of Sr is to refine and modify the morphology of Si particles. Ti and B were added in the form of Al-5Ti-B grain refiners. Fe is the impurity element in cast Al alloy, which can generate β-AlFeSi and α-AlFeSi phases with element Si. To eliminate the β-AlFeSi phase, Mn was added to generate α-AlFeMnSi particles. The addition of Mg can increase the mechanical properties by solution strengthening and generating strengthening phases like Mg_2_Si. The only variable in both alloys is the content of Sn, while other variables were controlled to be the same. Therefore, Al-9Si and Al-9Si-0.1Sn were listed for simplicity. 

These two alloys were separately melted in argon in a CXZG-0.5 (Shanghai Chen Xin Electric Furnace Co. Ltd., Shanghai, China) vacuum induction furnace from the raw materials that included high-purity (99.999 wt.%) Al particles, pure (99.99%) Mg particles, pure (99.9%) Sn particles (Φ2 × 5 mm), master alloys Al-20Si, Al-10Fe, Al-10Mn and modifying alloys Al-10Sr, as well as grain refiners Al-5Ti-B (all in wt.% unless specified). The heating current was 215 A and the heating time was 5 min. After holding for 5 min, the melt (from 680 to 720 °C) was poured into room-temperature graphite molds to form rods measuring Φ20 mm × 10 mm.

The tensile samples (shown in Figure S1 in the Supplementary Document) were wire-cut from the cast rods and then tested via an Instron 3369 testing machine at room temperature at a constant speed of 2 mm/min. A total of three samples of the same state were tested to obtain a datapoint. The side-on surfaces of tension-fractured samples were then ground, polished, and ultrasonically cleaned. The microstructures of the samples were observed via a Leica DM4500P (Leica Microsystems CMS GmbH, Wetzlar, Germany) optical microscope (OM) and a FEI Nova NanoSEM230 field emission gun scanning electron microscope (SEM) operating at 20 kV equipped with an energy dispersive spectrometer (EDS). The focused ion beam (FIB) machining was performed via a dual-beam scanning electron microscope (FEI Helios NanoLab G3 UC, Thermo Fisher Scientific Baltics, Vilnius, Lithuania) employing a Ga liquid metal ion source. Electron backscattering diffraction (EBSD) experiments were also performed on such an instrument with a scanning step of 6 μm. The distribution of cast defects was studied via 3-dimensional computed tomography (3DCT) on a Zeiss Xradia (Jena, Germany) 620 Versa instrument; the sample size was 2 × 1 × 5 mm with a resolution of 1.7 μm. The results were processed via Avizo 2019.1 software. The transmission electron microscopy (TEM) observations were performed via a Thermo Fisher (Waltham, MA, USA) Talos F200X field emission gun TEM operating at 200 kV. 

## 3. Results and Discussion

### 3.1. Grain Size

**Figure 1:**
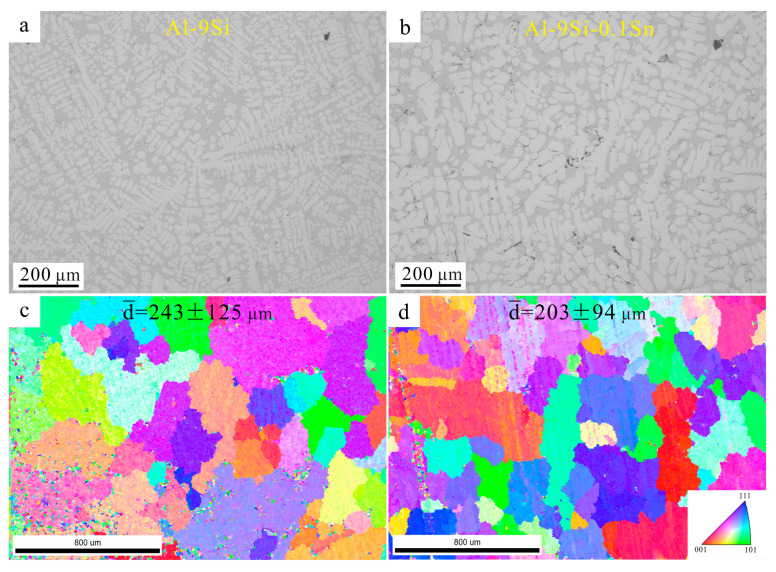
Microstructures of the (**a**,**c**) Al-9Si alloy and (**b**,**d**) Al-9Si-0.1Sn alloy. (**a**,**b**) OM images; (**c**,**d**) Inverse pole figure (IPF) maps.

Sn can slightly refine the grain size of Al-Si alloys, as shown in Figure 1. The brightest areas are α-Al phases. The eutectic Si phase is the grey contrast area. The black contrast areas are pores or holes generated during casting. To avoid the interference of low confidence interval (CI) index signals, the EBSD data were cleaned up via OIM analysis v7.3.1 software. Moreover, only the diameters of grains above 80 μm were counted to calculate the average diameters, which were 243 ± 125 μm and 203 ± 94 μm in the Sn-free and Sn-added samples, respectively. The grain size of the Al-9Si is slightly larger than that of Al-9Si-0.1Sn. However, according to the Hall–Patch equation [30,31], this small difference in grain size leads to a very slight change of yield stress of about 0.43 MPa. Therefore, the strengthening effect due to the refining of grain size via the addition of Sn is not discussed here.

### 3.2. Pores and Other Cast Defects Caused by Sn

**Figure 2:**
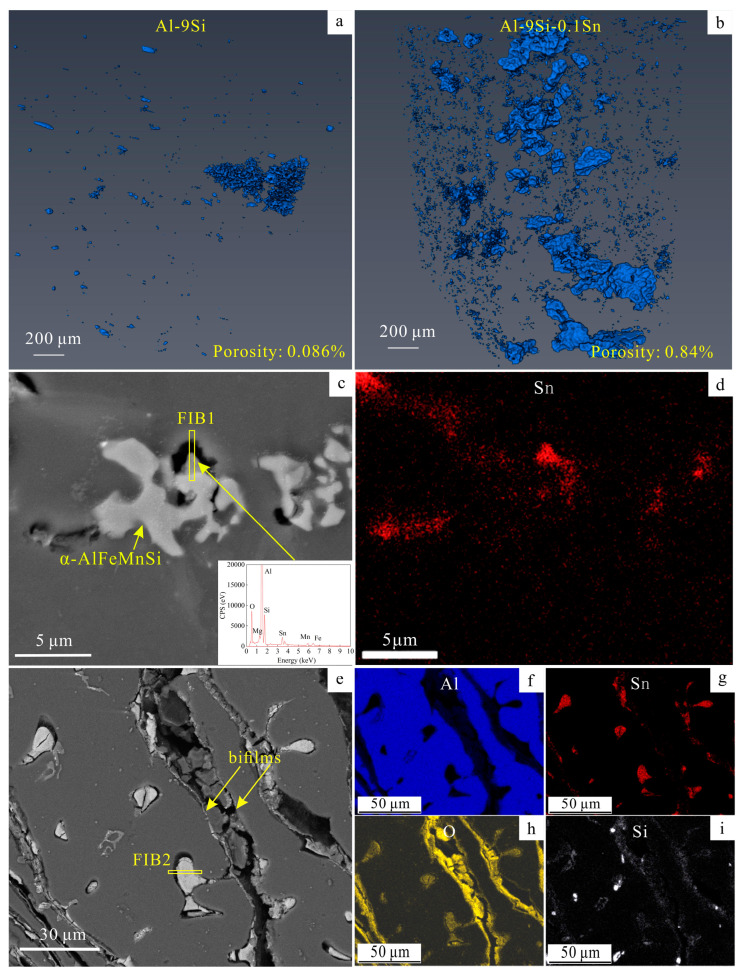
The morphology and distribution of casting defects. (**a**) The 3D distribution of casting defects in Al-9Si samples; (**b**) the 3D distribution of casting defects in Al-9Si-0.1Sn samples; (**c**) the SEM image of shrinkage pore defects in the Al-9Si-0.1Sn sample maps; (**d**) the Sn distribution of (**c**); (**e**) the SEM image of bifilm defects in the Al-9Si-0.1Sn sample; (**f**–**i**) EDS elemental maps of (**e**). EDS spectrum of white particles are shown in Figure S2.

Most Sn were found to be segregated near the oxide film, causing the obvious casting defects and the increase in porosity. Visualizing the distribution of defects in the current cast alloys using 3DCT, as shown in Figure 2a,b and Supplementary Videos S1 and S2, revealed that the porosity was increased by about 90% due to the Sn addition. Furthermore, as shown in Figure 2c,e, the segregation of Sn can be found around the holes in the cast rods with Sn addition according to the EDS mapping results. Especially, some large casting cracks were found, as shown in Figure 2e, and there were some white particles nearby. The corresponding EDS elemental maps show that the oxide layers (alumina bifilms [32]) lie on the cracks’ edges, in addition to some large white particles comprising O and Sn. 

Oxygen may come from the oxide films. During the melting process, the argon atmosphere and the alumina film on the surface protected the melt from being further oxidized. These alumina films were initially amorphous [33]. Then, with a low melting point, Sn tended to segregate at the areas of final solidification. When these areas were solidified near to alumina films, Sn could be oxidized due to the higher local contents of O near to the oxide films.

**Figure 3:**
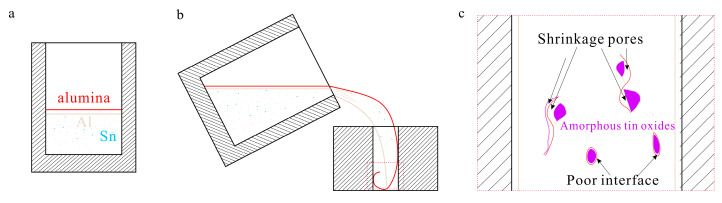
The schematic of shrinkage pore generation. (**a**) The state of alumina films during melting; (**b**) The state of alumina films during pouring; (**c**) The enlarged figure of the red rectangle in (**b**) after solidification. Red denotes alumina; yellow denotes aluminum; blue denotes Sn; purple denotes amorphous tin oxides.

The trapped liquids next to the enclosures were the main reason for the generation of shrinkage pores. The schematic is shown as Figure 3. The precursors of the oxide bifilms were the oxide films on the surface of the melt. When pouring the melt into the mold, the oxide films were rolled into the inner castings and broken into pieces through turbulence [32]. The Sn atoms appeared to segregate in the final solidification areas, causing the further decrease in local matrix solidification temperature [27]. Then, a quantity of liquid with a high Sn solution was trapped in the enclosures, which could not be refilled by other liquids. During solidification, some eutectic Si- and Fe-containing phases were induced to nucleate on the bifilm in the early stage [32]. The possibility of generating enclosed areas increased. The solidification of the remaining Sn-rich liquid caused shrinkage in the final state due to differences in the thermal expansion coefficients (10.05 × 10^−6^ °C^−1^ for tin dioxide [34] and 7.1 × 10^−6^ °C^−1^ for Alumina [35]). Therefore, shrinkage pores and poor interfaces were generated.

### 3.3. The Structure and the Composition of the Sn-Containing Particles

**Figure 4:**
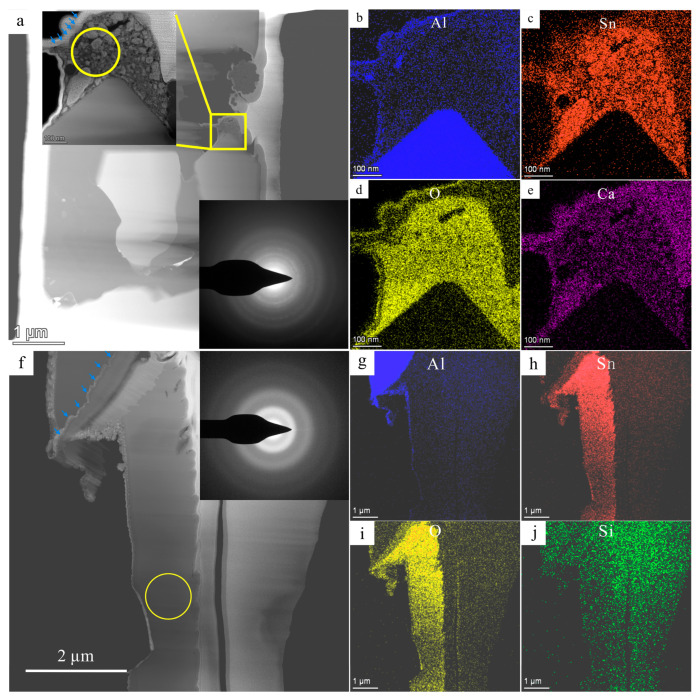
The morphology and element distribution of Tin oxides area in Figure 2c,e. (**a**) The HADDF-STEM image of FIB 1 sample (upper inset is the large figure in the yellow square; bottom inset is the SAED in the yellow circle of the upper inset); (**b**–**e**) EDS maps of upper inset in (**a**); (**f**) HADDF-STEM image of FIB 2 sample (inset is the SAED of the yellow circle); (**g**–**j**) the EDS maps of (**f**). The blue arrows denote alumina films. EDS spectra are shown in Figure S4.

FIB 1 and FIB 2 areas marked in Figure 2c,e were extracted and thinned via FIB, and then observed via TEM. According to the high-angle annular dark field (HADDF) images and corresponding EDS maps of FIB 1 shown in Figure 4c,e, Sn and Ca enriched the area in the yellow circle in Figure 4a. As a common impurity element in cast Al alloys, Ca always derives from residue slags in raw materials. Select area electron diffraction (SAED) shows that this area consisted of amorphous and polycrystalline materials. However, the signals of polycrystals were too weak to index, possibly due to some impurities containing oxides and calcium salts aggregating. The blue arrows in Figure 4a,f denote alumina films, proving that the tin oxides always exist near alumina films.

**Figure 5:**
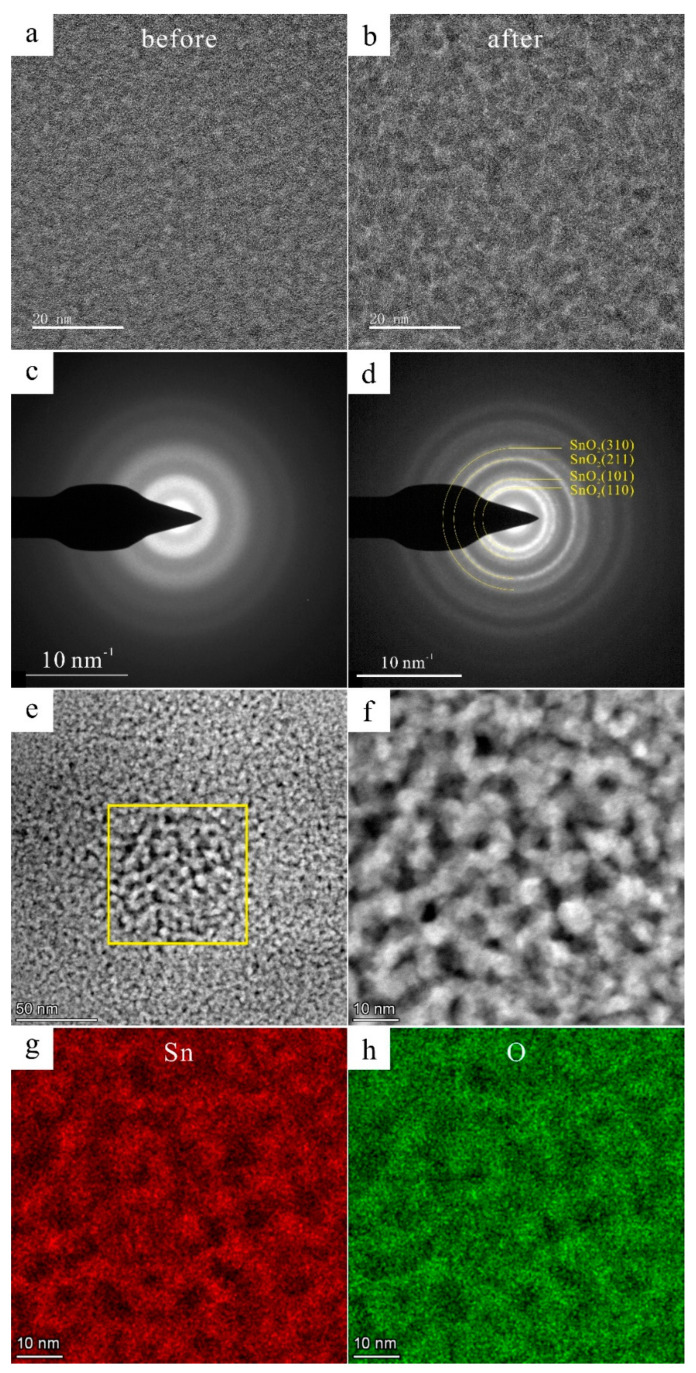
The crystallization of amorphous. (**a**) The HRTEM image before electron irradiation; (**b**) the HRTEM image after electron irradiation; (**c**) the SAED before electron irradiation; (**d**) the SAED after electron irradiation; (**e**,**f**) the HADDF-STEM images after electron irradiation; (**g**,**h**) the EDS mappings of Figure 4f.

The particles containing Sn and O were purely amorphous according to the TEM results shown in Figure 4f–j from the FIB 2 area. These amorphous materials were generated due to high cooling rates that suppressed the nucleation and growth of the crystallization [36]. The Sn^2+^ and Sn^4+^ may exist in these amorphous [37]. According to the high-resolution TEM (HRTEM) images and the corresponding fast Fourier transform (FFT) patterns shown in Figure 5a–d, after electron irradiation for seconds, the amorphous structure crystallized into polycrystalline rings. The HADDF-STEM and EDS maps also proved that the main elements were Sn and O. Therefore, these polycrystals were calibrated as SnO_2_ (PDF #46-1088, P42/mnm). Similar results have been reported in Zr_66.7_Cu_33.3_ alloys [38] and in Fe_85_B_15_ alloys [39] by other researchers.

Most of the added Sn content formed this amorphous phase according to an approximate calculation. Figure S3 is the schematic for calculating Sn concentration. The Sn concentrations obtained via overall EDS analysis (yellow regions in Figure S3a,c) of FIB 1 and FIB 2 were respectively 24.71 wt.% and 63.73 wt.%. Furthermore, the average concentration of Sn was 44.22 wt.%. Figure S3e presents the stacked pictures of the SEM images and the EDS maps of Sn. The red areas can approximately represent the distribution of Sn concentration after Gaussian blur. The total size of the three SEM areas was about 175,383 μm^2^. The size of the red areas was about 364 μm^2^. Therefore, the area fraction of these phases was calculated to be 0.21%. It was assumed that area fraction was equal to the volume fraction. The volume fraction and the average mass percentage of Sn were multiplied. The result was 0.09 wt.% Sn, which is close to 0.1 wt.%.

### 3.4. The Mechanical Behavior of the Casting Defects

**Figure 6:**
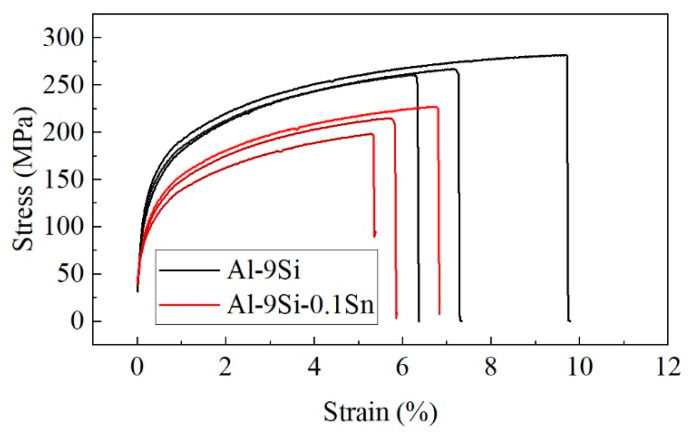
The stress-strain curves of Al-9Si and Al-9Si-0.1Sn samples.

Average tensile properties of Al-9Si and Al-9Si-0.1Sn samples are shown in Table 2. The stress-strain curves of all samples are shown in Figure 6. The results show that the ultimate tensile strength (UTS), yield strength (YS) and elongation (EL) were all diminished by adding Sn. 

**Figure 7:**
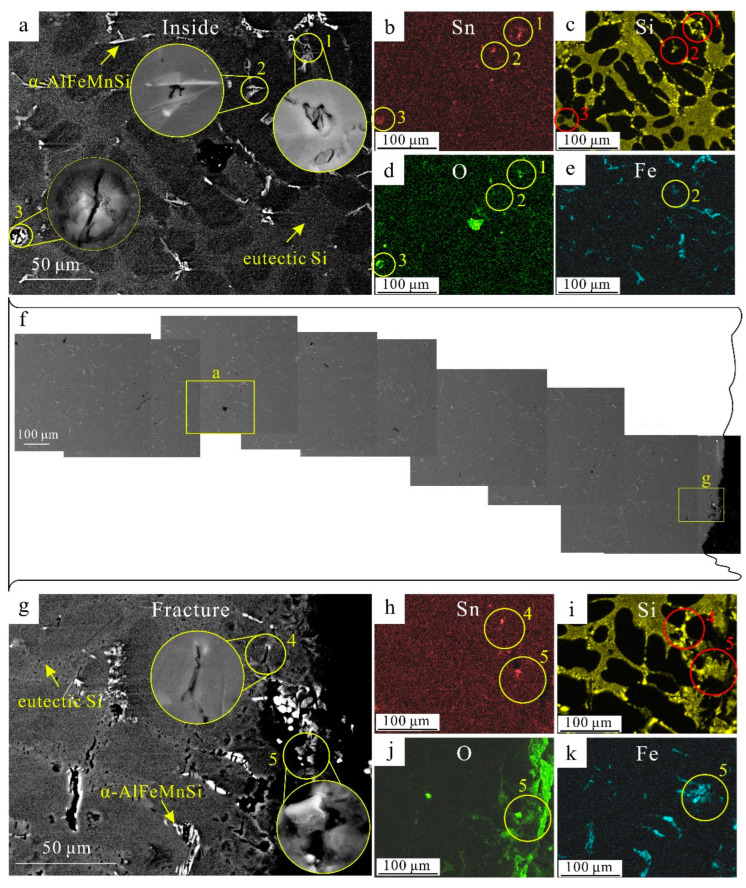
The BSE (backscattered electron) images and corresponding EDS maps of the surface of tensile Al-9Si-0.1Sn samples. (**a**) The BSE image far away from fracture; (**b**–**e**) the EDS maps of (**a**); (**f**) the schematic of tensile samples (more details are shown in Figure S5); (**g**) the BSE image in the front of the fracture; (**h**–**k**) the EDS maps of (**g**). EDS spectra are shown in Figure S6.

The casting defects initiated cracks during tensile deformation according to the SEM characterizations of fractured tensile samples shown in Figure 7. The EDS maps show the O and Sn were co-segregated in areas 1–5, marked in yellow circles in Figure 7a,g, which can be identified as tin oxides. The tin oxides in areas 1–3 were broken even though these areas were far away from the fracture which meant that it was easy for these tin oxides to be broken. This was because the ionic and covalent bonds in oxide amorphous materials were rigid and could not be switched as easily as is the case with metals [40]. In areas 4 and 5, at the front of fracture, the cracks were propagated along the tin oxides or the bifilms during tensile deformation. These fracture behaviors were similar to those of alumina [32]. 

Our research suggests not adding Sn to various cast Al alloys without heat treatments as these fatal casting defects, including the pores and brittle amorphous tin oxides, do not depend on any third element.

## 4. Conclusions

In this study, the fatal defects present in cast Al-Si alloys due to Sn addition have been investigated via multi-scale characterizations.

(1) Sn can significantly deteriorate both strength and elongation as it obviously increases porosity despite slightly refining the grain size.
(2) During casting, melt with high Sn solution was trapped in the enclosures. Shrinkage pores were then formed when cooling and amorphous tin oxides were generated near the alumina films.
(3) These casting defects including the pores and brittle amorphous tin oxides initiated cracks during tensile deformation.
(4) Our work suggests not adding Sn to various cast Al alloys without heat treatments as these fatal casting defects do not depend on any third element.

## Figures and Tables

**Table 1:** The alloys’ chemical compositions (wt.%).

Si	Mg	Fe	Mn	Sr	Ti	Sn	Al
9	0.22–0.26	0.09–0.15	0.17–0.24	0.011–0.024	0.05–0.14	0 or 0.1	Bal.

**Table 2:** The average tensile properties of the alloys.

Sample	UTS/MPa	YS/MPa	EL/%
Al-9Si	270 ± 9	144 ± 6	8 ± 1
Al-9Si-0.1Sn	214 ± 12	113 ± 6	7 ± 0

## Data Availability

Data will be made available on request.

## Supplementary Materials

The following supporting information can be downloaded at: https://www.mdpi.com/article/10.3390/ma16176020/s1, Figure S1: The size of tension samples; Figure S2: The crystallization of amorphous; Figure S3: The stress-strain curves of Al-9Si and Al-9Si-0.1Sn samples; Figure S4: The enlarged details information of Figure 4f in text; Figure S5: The enlarged details information of Figure 7f in text; Figure S6: The EDS spectrum of yellow circles in Figure 7a,g; Video S1: The 3D reconstruction of 3DCT on Al-9Si sample; Video S2: The 3D reconstruction of 3DCT on Al-9Si-0.1Sn sample [41,42].
