# Supplementary material for: The Fatal Defects in Cast Al-Si Alloys Due to Sn Addition
Source: Materials (Basel). 2023 Sep 1;16(17):6020. doi: 10.3390/ma16176020 (PMC10488968; doi:10.3390/ma16176020)
Supplement: Supplementary file 1 [file materials-16-06020-s001.zip › Supplementary Material V3.pdf]

Supplemental Material (SM)

The Fatal Defects in Cast Al-Si Alloys Due to Sn Addition

Yao Xiao [1], Jicheng Wang [2], Qianyu Deng [2], Li Feng [2], Dianming Peng [2], Hui Feng [3], Kai Li [1,4,*] and Yong Du [1,4]

Preparation of tension sample

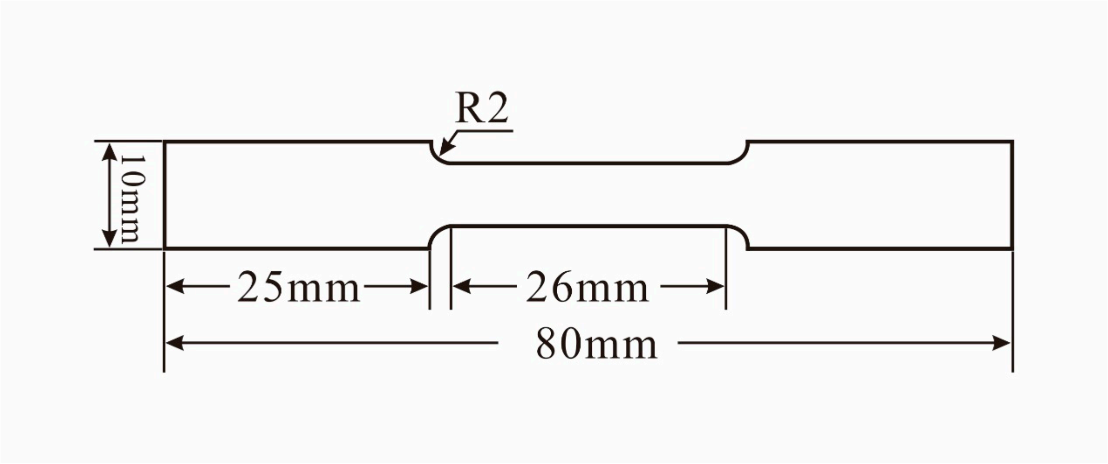

Figure S1 The size of tension samples

The EDS spectrum of while particles in Figure 2e

This is the EDS spectrum of the particle denoting as FIB2 in Figure 2e, which can be referred to the same particles

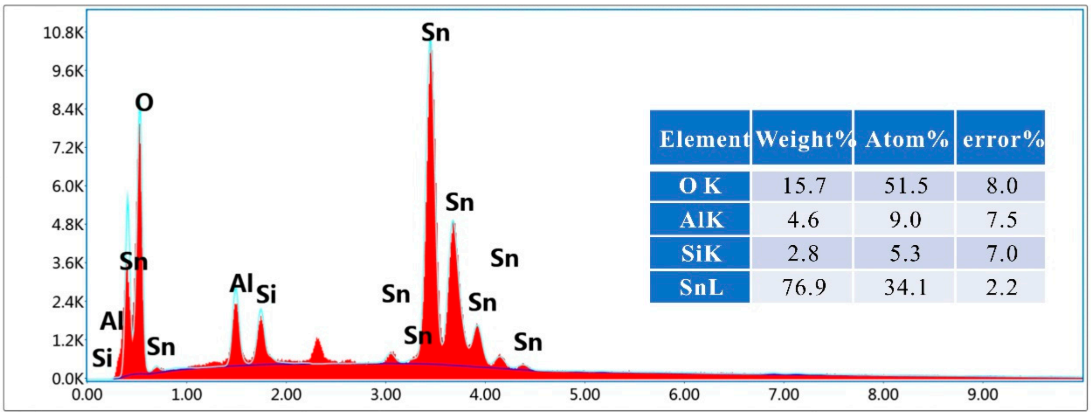

Element	Weight%	Atom%	error%
O K	15.7	51.5	8.0
AlK	4.6	9.0	7.5
SiK	2.8	5.3	7.0
SnL	76.9	34.1	2.2

Figure S2 The EDS spectrum of while particles in Figure 2e

The calculation of overall Sn contents to form amorphous phases

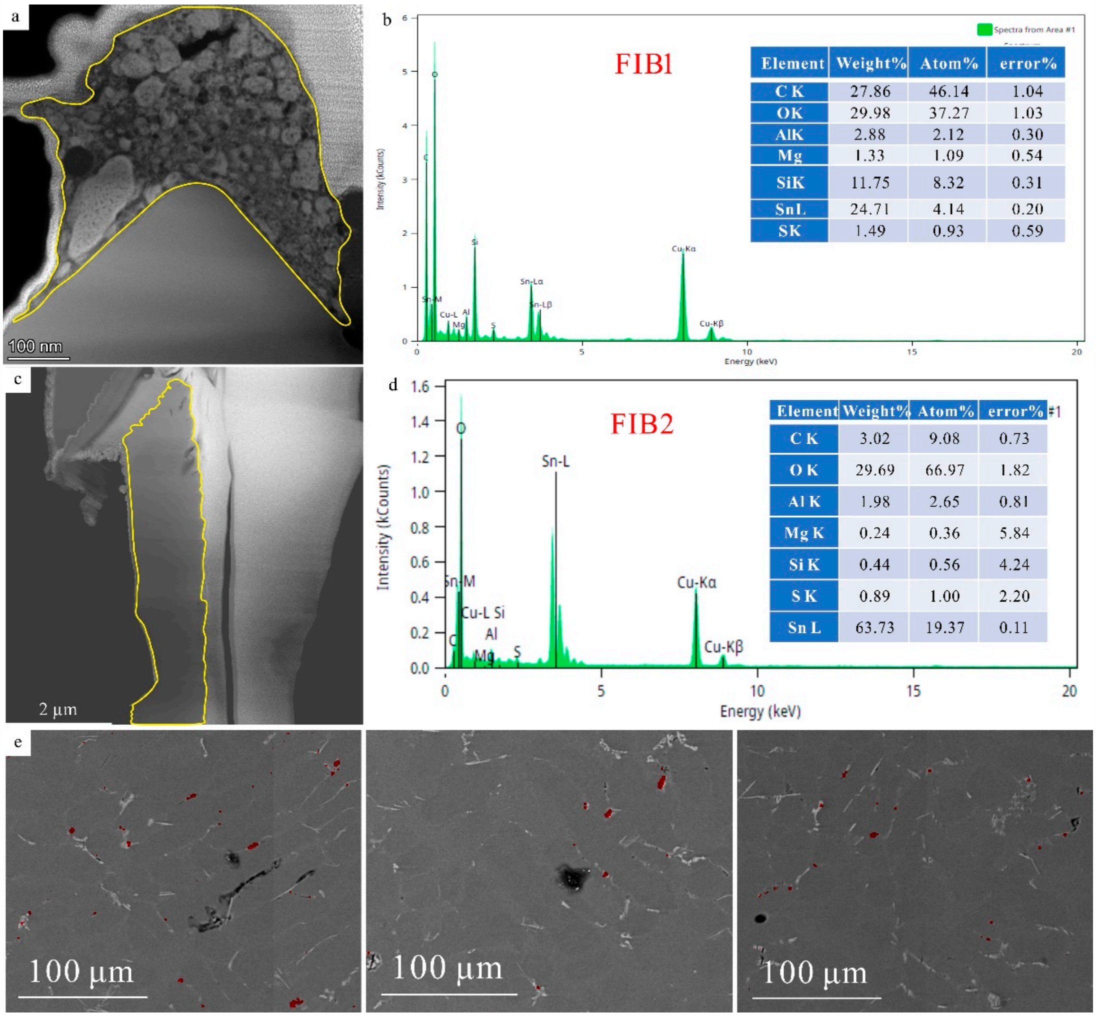

Figure **S3** The schematic of calculating Sn concentration. (a)The HADDF image of FIB 1 sample; (b) The EDS spectra of the yellow region in (a); (c)The HADDF image of FIB 2 sample; (d) The EDS spectra of the yellow region in (c); (e) the stacked pictures of the EDS maps of Sn and SEM.

The EDS spectra of FIB1 and FIB2

The EDS analysis in Figure S2 was acquired by SEM. According to the scattering of incident electron beam in the sample, the characteristic X-rays may come from a region larger than the beam. It means that the signals accounting for the 82% Al mainly came from Al matrix in actual fact, and the O mainly came from alumina and other oxides on sample surface. More accurate results are shown in Figure S4 of in the supplementary document, because the scattering of incident electron beam is reduced in thin TEM samples. The EDS of Figure S4(b) contains plenty of elements, because there were complex phases appearing in the yellow circle A in Figure S4(a). Shown below in Figure S4(c) is the EDS spectra of Sn-segregated areas in the same sample. Cu came from the copper mesh during FIB process. Al, Si and Sn came from the raw materials. C came from some organic materials deposited on the sample surface. Therefore, indeed, the O may not only bond with Sn, but also with Al, Ca and C.

Moreover, the EDS of Figure S4(e) is more accurate because the Sn segregated areas are all contained by yellow circle in Figure 4(f) of manuscript. In this EDS spectra, all elements except of Ca are the same. The Ca came from residue slags. The O element also bond with Sn, Al, Ca and C.

Secondary, the EDS results are not accurate enough when light (C, O) and heavy (Sn) elements are analyzed together [1]. There are fewer orbital electrons in atoms of light elements, so that their characteristic X-rays are limited. Sometimes the L peaks of heavy elements overlap with the K peaks of light elements [2]. The lower signal-noise ratio makes the quantification of contents of light elements with larger errors. Therefore, the measured ratio between O and Sn is deviated from 2:1. However, the evidence of SnO_2 is the FFT in Figure 5(d). In other words, when an amorphous-to-crystal transformation occurs due to electron radiation as shown in Figure 5, the crystal structure formed is in agreement with that of crystalline SnO_2. Therefore, we would like to call the oxides as amorphous Sn-rich oxides, which probably contain SnO_2.

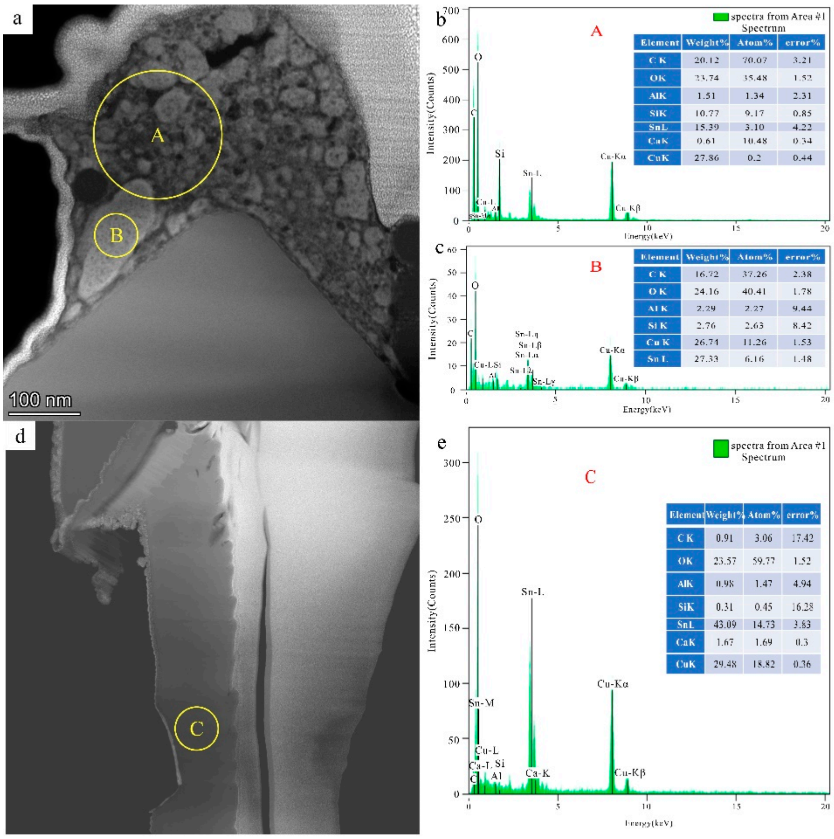

Figure S4 The HADDF-STEM images and EDS spectrum of yellow circles of FIB 1and FIB 2. (a) HADDF image of FIB 1; (b) EDS spectra of the yellow circle A; (c) EDS spectra of the yellow circle B; (d) HADDF image of FIB 2; (e) EDS spectra of the yellow circle C.

The contribution of Sn in tensile samples

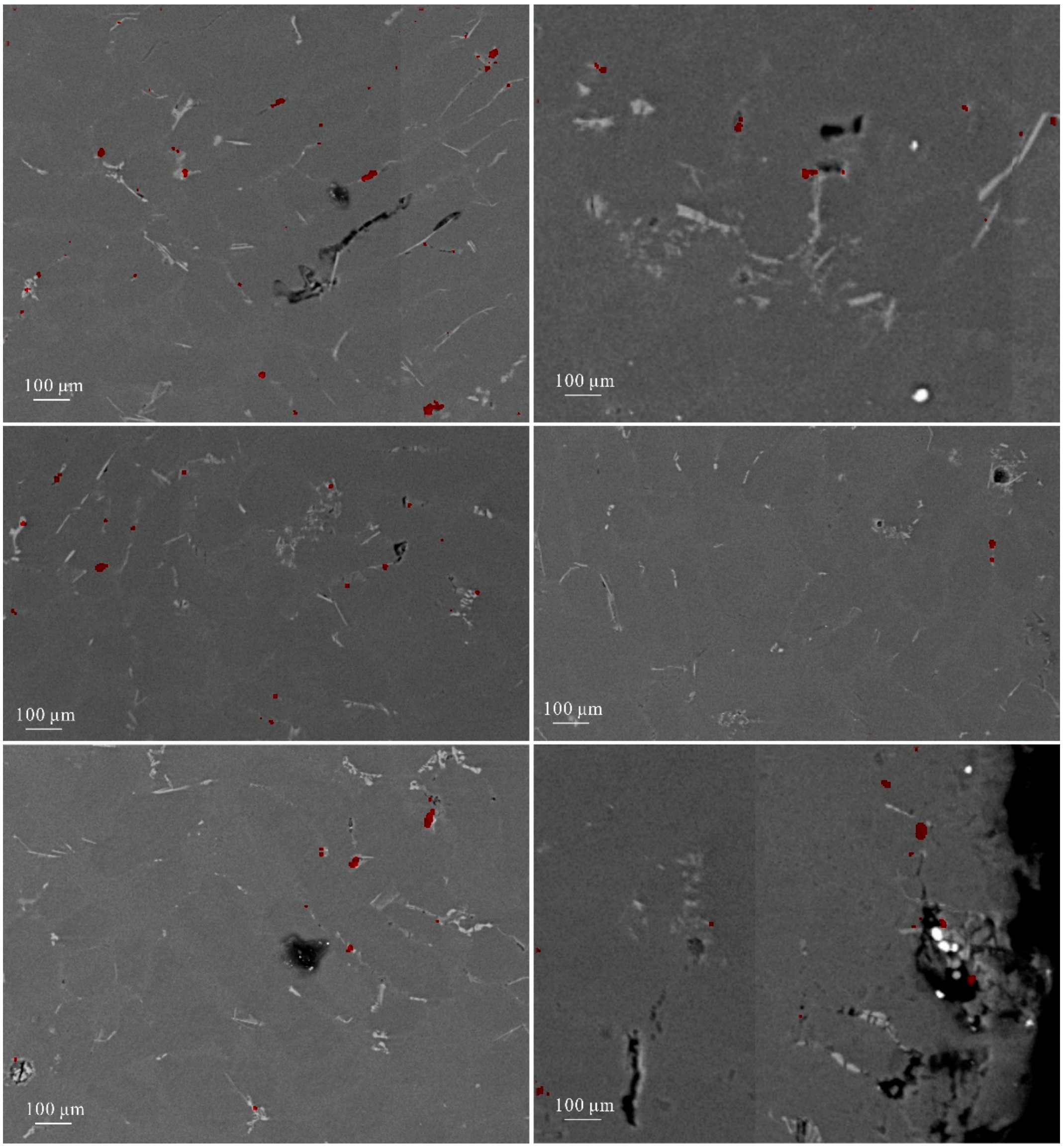

Figure S5 The enlarged details information of Figure 7f in text.

The EDS spectrum of yellow circles in Figure 7

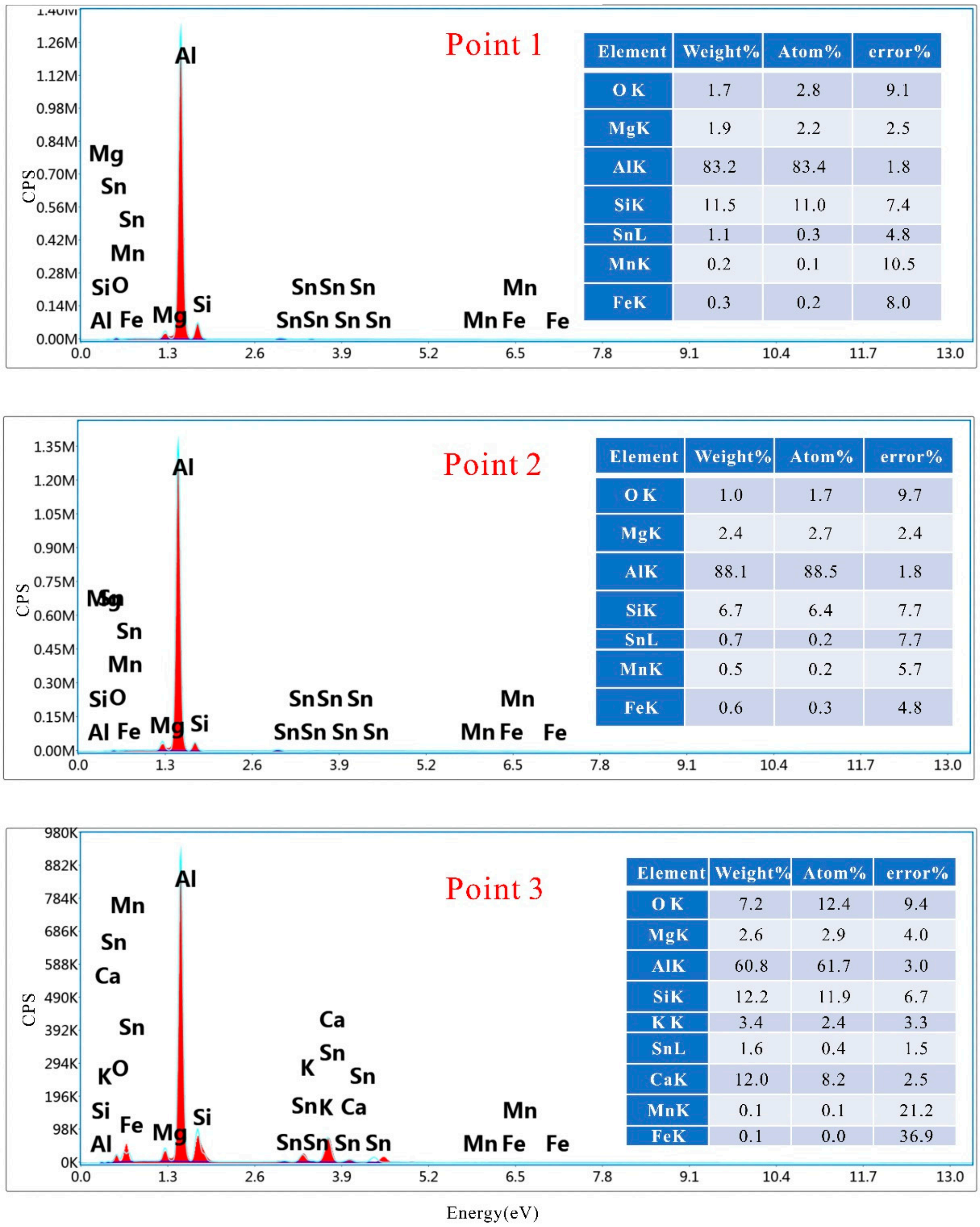

Point 1

Element	Weight%	Atom%	error%
O K	1.7	2.8	9.1
MgK	1.9	2.2	2.5
AlK	83.2	83.4	1.8
SiK	11.5	11.0	7.4
SnL	1.1	0.3	4.8
MnK	0.2	0.1	10.5
FeK	0.3	0.2	8.0

Point 2

Element	Weight%	Atom%	error%
O K	1.0	1.7	9.7
MgK	2.4	2.7	2.4
AlK	88.1	88.5	1.8
SiK	6.7	6.4	7.7
SnL	0.7	0.2	7.7
MnK	0.5	0.2	5.7
FeK	0.6	0.3	4.8

Point 3

Element	Weight%	Atom%	error%
O K	7.2	12.4	9.4
MgK	2.6	2.9	4.0
AlK	60.8	61.7	3.0
SiK	12.2	11.9	6.7
K K	3.4	2.4	3.3
SnL	1.6	0.4	1.5
CaK	12.0	8.2	2.5
MnK	0.1	0.1	21.2
FeK	0.1	0.0	36.9

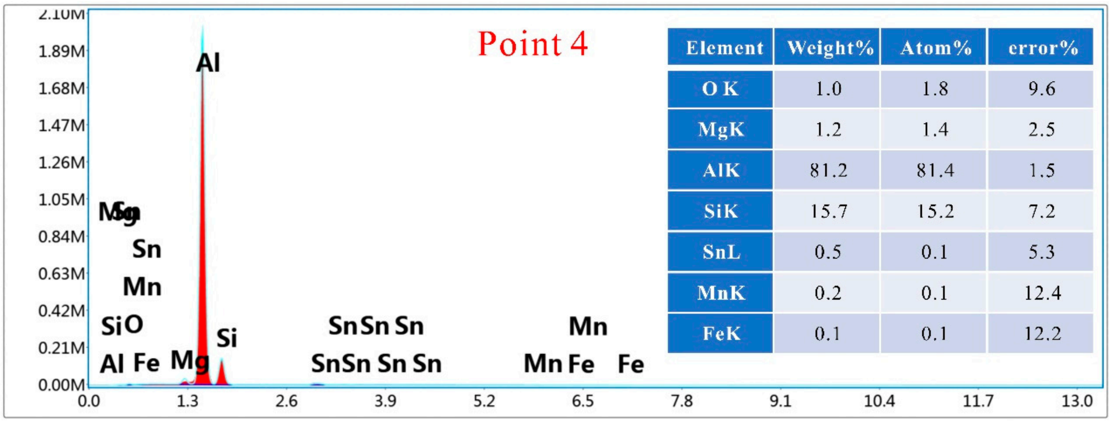

Element	Weight%	Atom%	error%
O K	1.0	1.8	9.6
MgK	1.2	1.4	2.5
AlK	81.2	81.4	1.5
SiK	15.7	15.2	7.2
SnL	0.5	0.1	5.3
MnK	0.2	0.1	12.4
FeK	0.1	0.1	12.2

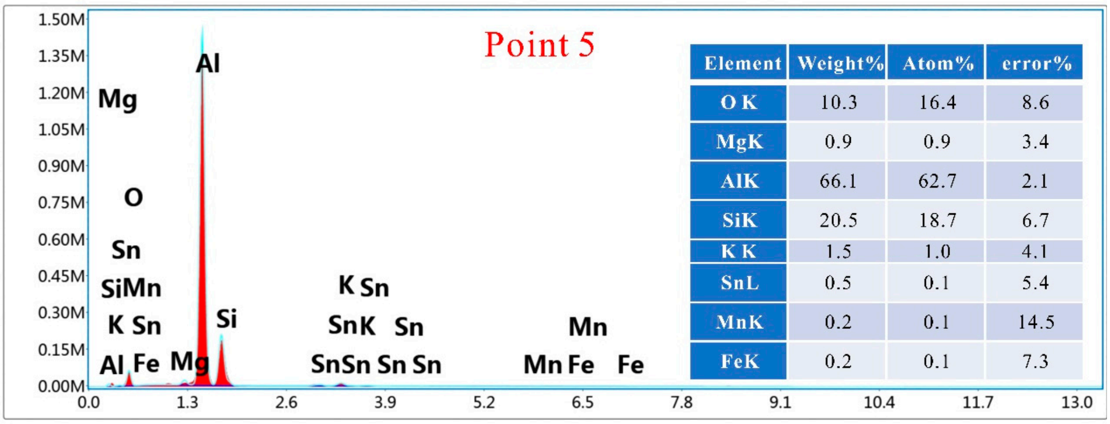

Element	Weight%	Atom%	error%
O K	10.3	16.4	8.6
MgK	0.9	0.9	3.4
AlK	66.1	62.7	2.1
SiK	20.5	18.7	6.7
K K	1.5	1.0	4.1
SnL	0.5	0.1	5.4
MnK	0.2	0.1	14.5
FeK	0.2	0.1	7.3

Figure S6 The EDS spectrum of yellow circles in Figs.7a and 7g.

Reference

[1] Osán, J.; De Hoog, J.; Van Espen, P.; Szalóki, I.; Ro, C.U.; Van Grieken, R. Evaluation of energy-dispersive x-ray spectra of low-Z elements from electron-probe microanalysis of individual particles. *X-Ray Spectrometry: An International Journal* **2001**, *30*, 419-426.

[2] Carter, D.B.W.C.B. *Transmission Electron Microscopy A Textbook for Materials Science*; springer publication: 2009.
